# Reliability of the Second and Third Iterations of the Sensory–Motor Dysfunction Questionnaire in a Subclinical Neck Pain Population

**DOI:** 10.3390/brainsci15010067

**Published:** 2025-01-14

**Authors:** Ushani Ambalavanar, Heidi Haavik, Nitika Kumari, Imran Amjad, Nooshin Khobzi Rotondi, Bernadette Ann Murphy

**Affiliations:** 1 Faculty of Health Sciences, Ontario Tech University, Oshawa, ON L1G 0C5, Canada; 2 Center of Chiropractic Research, New Zealand College of Chiropractic, 6 Harrison Road, Mount Wellington, Auckland 1060, New Zealand

**Keywords:** spine pain, sensorimotor integration, self-report questionnaire, reliability, internal consistency

## Abstract

Background/Objectives: This study aimed to address limitations of the pilot reliability study on the Sensory–Motor Dysfunction Questionnaire (SMD-Q) in two parts. Part 1 evaluated the intra-rater reliability of SMD-Q version 2 (V2). Part 2 addressed V2’s limitations before assessing the intra-rater reliability of version 3 (V3). V2 framed questions as “over the past week”, whereas V3 also framed questions as “in a typical/usual week”. Methods: The SMD-Q was administered via Qualtrics^TM^ at baseline and 4 to 7 days later to subclinical neck pain participants, 51 in part 1 (32 F; mean age ± SD: 21.17 ± 2.66 y) and 27 in part 2 (20 F; mean age ± SD: 21.89 ± 2.81 y). Reliability statistics (quadratic weighted kappa (K_w_) and Cronbach’s alpha (α)) were calculated for all items (V2) and total scores (V2 and V3). Results: There was excellent agreement for V2 total scores (K_w_ ≥ 0.75), and V3 total scores for “in a typical/usual week” (K_w_ ≥ 0.75), but fair to good agreement for V3 total scores of “over the past week” (0.40 < K_w_ < 0.75). V2 had acceptable (0.7 ≤ α < 0.8) to good internal consistency (0.8 ≤ α < 0.9), while V3 had good internal consistency for both administrations. Conclusions: Versions 2 and 3 of the SMD-Q appear to reliably capture disordered sensorimotor integration in people with subclinical neck pain, with improved reliability in V3 when questions are framed as “in a typical/usual week”. However, further research is needed to confirm this finding.

## 1. Introduction

Musculoskeletal pain of any severity or chronicity that presents in conjunction with vertebral column dysfunction has been found to result in disordered sensorimotor integration and multi-modal integration [1]. The symptoms can vary among individuals, ranging from alterations in central and subcortical somatosensory processing [2,3,4,5,6], multi-modal integration (MMI) [7,8], motor control [9], limb sensorimotor integration (SMI), and motor performance [10,11,12,13], to sensorimotor function [14,15,16,17,18,19,20,21,22]. The transition from acute, recurrent/subclinical to chronic musculoskeletal pain has been theorized to be the result of deep paraspinal activity [1,23], which has not been captured with a patient-reported outcome measure (PROM). As there has not been a PROM that documented and/or quantified the subjective manifestations of altered SMI and MMI that might accompany recurrent or chronic spinal problems, a PROM was developed by Ambalavanar et al. [24].

The PROM developed by Ambalavanar et al. [24], known as the Sensory–Motor Dysfunction Questionnaire (SMD-Q), filled the gap in the literature regarding neurophysiological disruptions from spine dysfunction, i.e., subclinical or chronic. The pilot version of the SMD-Q appears to reliably capture disordered SMI and MMI in individuals with subclinical neck pain; however, that study suggested the questionnaire be revised and administered to a sample size > 30, to confirm those findings [24]. The second iteration of the SMD-Q (SMD-Q version 2, with recommendations implemented) can discriminate healthy participants from individuals with neck pain (i.e., subclinical or chronic) and may be sensitive to changes in response to treatment in individuals with neck pain [25]. It is unknown whether the second iteration of the SMD-Q is reliable in individuals with subclinical neck pain.

This study builds on the preliminary version of a novel questionnaire designed to quantify disordered SMI in individuals with spinal pain. The purpose of the first part of this study was to assess the reliability of the second iteration of the SMD-Q in a population with subclinical neck pain (same as a previous reliability study [24]). The objective of part 1 of this study was to determine whether the implementation of the recommendations made in the previous reliability study improved the reliability of the individual items and the total score of the SMD-Q. The purpose of the second part of this study was to assess the reliability of the third iteration of the SMD-Q (SMD-Q version 3), which was developed in response to the recommendations made following part 1 of this study. The objective was to investigate whether framing questions as “in a typical/usual week” versus “over the past week” improved the SMD-Q’s reliability with this subclinical population. This recommendation is valuable as it can capture the variability in pain episodes within this population, where variability can confound reliability outcome measures.

## 2. Materials and Methods

### 2.1. Participants

Undergraduate-aged students (18–35 years of age) attending Ontario Tech University (OnTechU) were invited to participate in this study. Participants were recruited through online course announcements or emails sent through the university’s communication system. The recruitment period was from 7 September 2021 to 30 March 2022, and from 12 September 2022 to 21 April 2023, for part 1 and part 2, respectively.

Individuals with subclinical neck pain (SCNP) were eligible to participate in this study. The inclusion criteria for SCNP were as follows: (1) participants experienced recurrent and ongoing episodes of neck pain, aches, stiffness, or discomfort, and (2) had not sought treatment for this dysfunction in the past month [26,27]. This population often presents with other regions of spinal pain alongside their intermittent neck problems. Hence, those individuals were included if they fit the criteria of SCNP and had not sought treatment for other regions of spinal pain. The exclusion criteria were self-reported symptoms: (a) those associated with a formal diagnosis of a neurological condition (e.g., head injury/concussion, etc.) known to impact the central processing of somatosensory information, and (b) other pain conditions (e.g., dental pain, abdominal pain, or visceral pain, etc.) that could influence sensorimotor function.

Eligibility was confirmed by the Von Korff Chronic Pain Grade Scale, which determined whether interested participants had SCNP, and documented their degree of pain-related functionality [28]. SCNP participants who had a pain grade between I (low disability—low intensity) and IV (high disability—severely limiting) were deemed eligible [28,29]). Participants were ineligible to complete the second administration of the questionnaire (4 to 7 days later) if they acquired an event that may have impaired or impacted their cognitive function and/or central processing (e.g., spine or brain injury) after the baseline administration. A range of within a week was chosen for the second administration as recurrent pain episodes can vary from week to week; as a result, a shorter recall period is needed to capture disordered SMI and MMI on sensorimotor function in the subclinical spine pain population and minimize recall error [30].

Given that part 2 of this study was focused on the reliability of the revisions/suggestions made in response to part 1, a sample size less than 30 was deemed sufficient for a proof of principle [31]; i.e., it reveal trends in which revisions/suggestions worked or did not work.

Informed consent was obtained electronically, where participants clicked “I agree” after reading the consent form and before starting the SMD-Q. This study was approved and conducted in accordance with the OnTechU Research Ethics Board (File #:16317).

### 2.2. The Sensory–Motor Dysfunction Questionnaire

The SMD-Q consists of 12 items quantifying the frequency of poor sensorimotor function, to gauge the degree of sensorimotor dysfunction [24]. The second iteration of the SMD-Q implemented the suggestions made in the pilot study, which included the removal or rephrasing of questions with low agreement, breaking up of a question with multiple items, and the inclusion of more concrete examples for questions with fair to good agreement [24]. The 12 items were framed as “over the past week” in the second iteration of the SMD-Q (see Appendix A), while the third iteration of the SMD-Q also posed the questions as “in a typical/usual week” (see Appendix B). The third iteration of the SMD-Q permits the comparison of “in a typical/usual week” vs. “over the past week”.

Participants were instructed to choose the option that best described their motor behavior. The options included the following: (1) never/rarely occurs when performing this action/task (<1 day); (2) occurs some or little of the time when performing this action/task (1–2 days of the week); (3) often or a moderate amount of time when performing this action/task (3–4 days of the week); and (4) most or all of the time when performing this action/task (5 or more days of the week). The categorical response was transformed into a numerical value, from 0 to 3. The sum of the numeric value of the categorical option chosen for each question equated the total score of the SMD-Q, where the maximum score is 36. A higher score is indicative of a greater degree of dysfunction, while a lower score is indicative of a lower degree of dysfunction.

### 2.3. Pain Visual Analog Scale (VAS)

A pain VAS was also administered in Qualtrics™ prior to completing the SMD-Q, to measure the intensity of pain on the day of testing. This question was presented as a slider with zero (no pain) on the far-left side and 100 (worst pain experienced) on the far-right side of the 400-pixel (∼100 mm) horizontal scale. The location of the participant’s cursor on the scale was quantified by Qualtrics™ software (Qualtrics, Provo, UT, USA. https://www.qualtrics.com/), version September 2021–April 2023.

### 2.4. Experimental Protocol

In part 1 and part 2 of the study, the SMD-Q was administered to SCNP participants using the Qualtrics™ software by the researcher at baseline and day 4, but participants had until day 7 to complete the questionnaire. Participants completed the second administration of the SMD-Q in a single session. This range was chosen as it would allow for better recall [30] and minimize variability in scores since symptom presentation would be more uniform in an SCNP population.

### 2.5. Statistical Analysis

The individual and total scores were assessed for test–retest reliability of the second iteration of the SMD-Q, but the total score was only assessed for test–retest reliability for the third iteration of the SMD-Q. The individual items were assessed for the second iteration of the SMD-Q, to confirm the revisions implemented yielded improvements in reliability statistics, which would also be captured in the total score of the SMD-Q. The investigation of the total score in the third iteration of the SMD-Q was performed because subclinical spine pain leads to an array of symptomology from individual to individual. In other words, not all individuals with subclinical spine pain will exhibit each subjective manifestation of altered SMI and MMI. If that part of the central processing is not impaired and/or being overcompensated by the central nervous system, then it will not be captured in the individual items. Therefore, the assessment of the total score permits the investigation in the degree of sensorimotor dysfunction across individuals that vary in symptom presentation.

All tests (normality, reliability statistics, and correlations) were performed using IBM SPSS Statistics version 27.0 (IBM Corp. Released 2020. IBM SPSS Statistics for Windows, Version 27.0. Armonk, NY, USA: IBM Corp) [32].

#### 2.5.1. Part 1: Second Iteration of the SMD-Q

The Kolmogorov–Smirnov test (n > 50) revealed that the individual item scores and the total score were non-normally distributed at both timepoints.

#### 2.5.2. Part 2: Third Iteration of the SMD-Q

The Shapiro–Wilk test (n < 50) demonstrated that the total score was non-normally distributed at both timepoints, for both “in a typical/usual week” and “over the past week”.

#### 2.5.3. Test–Retest Reliability

As the datasets in part 1 (item and total score) and part 2 (total score) of the study were non-normally distributed, the weighted kappa (K_w_) was calculated and reported [33]. The cut-offs and interpretations proposed by Fleiss et al. [34] were used, i.e., K_w_ ≤ 0.40 (poor agreement beyond chance), 0.40 < K_w_ < 0.75 (fair to good agreement beyond chance), and K_w_ ≥ 0.75 (excellent agreement beyond chance). The 95% confidence intervals (CIs) were also reported.

#### 2.5.4. Internal Consistency

The internal consistency of the second and third iterations of the SMD-Q were assessed at baseline and at the second administration using Cronbach’s alpha (α), which is an indicator of reliability. The cut-offs and interpretations proposed by George and Mallery [35] were used, where an α < 0.5 is considered unacceptable, 0.5 ≤ α < 0.6 is considered poor, 0.6 ≤ α < 0.7 is considered questionable, 0.7 ≤ α < 0.8 is considered acceptable, 0.8 ≤ α < 0.9 is considered good, and α ≥ 0.9 is considered excellent.

#### 2.5.5. Correlations

Correlations between the pain VAS score and the total SMD-Q at baseline, as well as at the second administration, were conducted for the second and third iterations of the SMD-Q. This analysis provides insight into whether the reliability statistics of the total scores of the SMD-Q were influenced by changes in symptom presentation.

Spearman’s rho correlations with two-tailed significance were conducted as the pain VAS scores and the total scores were non-normally distributed at each timepoint and for each iteration of the SMD-Q. Statistical significance was set at *p* < 0.05.

## 3. Results

### 3.1. Part 1: Reliability Testing of the Second Iteration of the SMD-Q

#### 3.1.1. Spine Pain Characteristics

Fifty-six participants met the eligibility criteria and completed the questionnaire at baseline and the second administration, where no data were missing. Five datasets were excluded from the statistical analysis because the second administration of the SMD-Q was completed after the seventh day. Table 1 provides the descriptive and pain characteristics of the sample.

#### 3.1.2. Test–Retest Reliability and Internal Consistency of Second Iteration of the SMD-Q

Five items had poor agreement beyond chance (K_w_ ≤ 0.40) [34], seven items had fair to good agreement beyond chance (0.40 < K_w_ < 0.75) [34], and the total score had excellent agreement beyond chance (K_w_ ≥ 0.75) [34]; see Table 2 for the K_w_ value and the 95% CI. The internal consistency of the SMD-Q was acceptable (α = 0.748) at baseline, and good (α = 0.856) at the second administration.

#### 3.1.3. Spearman Correlation of Second Iteration of the SMD-Q

There was a significant positive correlation between the SMD-Q total score and pain VAS score at baseline (r_s_ = 0.303, *p* = 0.036), and at the second administration (r_s_ = 0.389, *p* = 0.005).

### 3.2. Part 2: Reliability Testing of the Third Iteration of the SMD-Q

#### 3.2.1. Spine Pain Characteristics

We aimed to recruit thirty participants, but only twenty-seven participants expressed interest and met the eligibility criteria. The twenty-seven participants with SCNP completed the third iteration of the SMD-Q at the respective timepoints; see Table 3 for descriptive and pain characteristics of the sample.

#### 3.2.2. Test–Retest Reliability and Internal Consistency of the Third Iteration of the SMD-Q: Total Scores

There was fair to good agreement beyond chance (0.40 < K_w_ < 0.75) for the total score of items framed as “over the past week”. There was excellent agreement beyond chance (K_w_ ≥ 0.75) for the total score of items framed as “in a typical/usual week”. See Table 4 for the weighted kappa statistics and the 95% CI. The 12 items framed as “over the past week” had good internal consistency (0.8 ≤ α < 0.9) at baseline (α = 0.881), and at the second administration (α = 0.838). The 12 items framed as “in a typical/usual week” had good internal consistency at baseline (α = 0. 895), and at the second administration (α = 0. 869).

#### 3.2.3. Spearman Correlation of the Third Iteration of the SMD-Q

There was no significant correlation between the pain VAS scores and SMD-Q scores of items posed as “over the past week”, at baseline (r_s_ = 0.003, *p* = 0.990), and at the second administration (r_s_ = 0.304, *p* = 0.124). There was no significant correlation between the pain VAS scores and SMD-Q scores of items posed as “in a typical/usual week”, at baseline (r_s_ = −0.131, *p* = 0.516), and at the second administration (r_s_ = 0.067, *p* = 0.740).

## 4. Discussion

Part 1 of this study demonstrated that implementing the recommendations suggested by Ambalavanar et al. [24], which included removing or rephrasing items with poor agreement (K_w_ ≤ 0.40), breaking up multi-part questions, and adding more concrete example for items with a K_w_ between 0.4 and 0.75, improved the reliability of the SMD-Q’s total score. Part 1 of this study demonstrated a positive correlation between the pain VAS scores and the SMD-Q total score, suggesting that the symptom change may have influenced the self-reporting of sensorimotor dysfunction, which led to an increased reliability of the SMD-Q. The improved reliability statistic of the total score fulfilled one of part 1’s objectives; however, the objective of improving each individual item (i.e., K_w_ > 0.75) was not fulfilled. Five items had poor agreement, and the remaining seven items had fair to good agreement. This variation led to the suggestion of posing questions as “in a typical/usual week”, which resulted in the third iteration of the SMD-Q. The objective of improving the reliability of the total score in the third iteration of the SMD-Q was accomplished via part 2 of this study. In part 2, it was found that the total score of items framed as “in a typical/usual week” led to excellent reliability and greater internal consistency at both timepoints versus when items were framed as “over the past week”. Part 2 of this study demonstrated no correlation between the pain VAS scores and the SMD-Q total scores at both administrations, suggesting that changes in pain did not directly impact reliability statistics regardless of whether “over the past week” or “in a typical/usual week” wording is used. This finding suggests that the use of “in a typical/usual week” may be appropriate for a subclinical spine pain population whose symptom presentation varies in degree, but further testing is needed to confirm these findings.

### 4.1. Part 1: Reliability of Individual Items and Total Score

Items reflecting constructs of hand–eye coordination (Q2), head and neck proprioception (Q3), full-body proprioception (excluding head and neck) (Q4), failed during grasping motor tasks (Q6), misstepped (Q7), and MMI (Q12) had K_w_ values that were consistent with the previous reliability study; i.e., they remained in the same K_w_ cut-offs [24]. This suggests that the changes implemented in response to the pilot reliability study led to similar results, reliably assessing the aforementioned constructs of SMI in this subclinical population. The increases in the K_w_ values on the constructs of postural control (Q1) and the uni-sensory processing of auditory stimuli (Q9) reveal an improvement in reliability, reflective of the changes in the phrasing of questions [24].

Constructs of missed during reaching motor tasks (Q5), motor performance (Q8), uni-sensory processing of visual stimuli (Q10), and bombardment of sensory stimuli (Q11) had lower K_w_ values than the pilot reliability study [24]. Item 11 was not revised following the pilot reliability study; however, the current fair to good agreement beyond chance suggests this sample had greater variability in this construct, seen as inconsistencies in responses between baseline and 4 to 7 days later. Although the recommended changes were implemented (e.g., wording and adding more concrete examples) for questions 5, 8, and 10, the changes in the weighted kappa statistic could be the result of inconsistent responses since SCNP participants experienced intermittent episodes.

The Cronbach’s alpha values differed from the previous reliability study [24]. There is acceptable internal consistency at baseline and good internal consistency at the second administration for part 1 of this study, while the previous reliability study had excellent internal consistency at both timepoints [24]. Part 1 of this study had a larger sample size and a variable endpoint (second administration 4 to 7 days after the first), while the previous reliability study had a smaller sample (<30) but a uniform 7-day interval for the second administration. It is plausible that changes made in response to the recommendations proposed by Ambalavanar et al. [24] could also account for the acceptable internal consistency at baseline. The improvement in internal consistency at the second administration could be the result of good recall or reduced variability in motor behavior from a lack of intermittent episodes within the one-week time frame [36]. The theory of reduced variability in motor behavior impacting the reliability statistics is strengthened by the positive correlation between the pain VAS score and the total score.

### 4.2. Suggestions for Third Iteration of the SMD-Q

The recommendations made in the previous reliability study, which were implemented in part 1 of the study, led to a total score that had excellent agreement beyond chance, and revealed the SMD-Q had acceptable to good internal consistency. However, the weighted kappa statistic of the individual items varied between the previous study and part 1 of this study, demonstrating disparities in sensorimotor function among individuals with subclinical spine pain. Given that the frequency and intensity of pain episodes vary in those with SCNP, it is also expected that the impact of SCNP on different constructs of SMI might also vary in degree. It is recommended that future questions are framed as “in a typical/usual week” as opposed to “in the past week”. This change in phrasing could better capture the typical/usual level of impairment in those constructs without being confounded by the variability in pain within a subclinical pain population.

### 4.3. Part 2: Reliability of Total Scores (In a Typical/Usual Week vs. Over the Past Week)

The total score for “in a typical/usual week” had excellent agreement beyond chance, whereas the total score for “over the past week” had fair to good agreement beyond chance. The weighted kappa statistic of the total score for “over the past week” from part 2 (K_w_ = 0.74; fair to good agreement) differed from part 1 (K_w_ = 0.82; excellent agreement), which could be because of sample size differences, i.e., > 30 for part 1 versus < 30 for part 2. The weighted kappa statistic for total score of items framed as “in a typical/usual week” (K_w_ = 0.830) was slightly higher than the weighted kappa statistic for part 1’s “over the past week” total score (K_w_ = 0.820), both showing excellent agreement beyond chance. Although the sample size varied between part 1 and part 2 of this study, framing the question as “in a typical/usual week” yielded a slightly better weighted kappa value. These findings suggest that the use of “in a typical/usual week” may yield more consistent responses in an SCNP population, which is also reflected by the greater and consistent Cronbach’s alpha value. This is supported by a change in direction (negative to positive) and reduction in the strength (weak to weaker) of the correlation between the pain VAS scores and the total score of items worded as “in a typical/usual week” from the second administration to baseline.

#### Limitation

The small sample size was a limitation for part 2 of this study. A minimum sample size of 30 [37] or 32 [38] is needed to obtain a precise estimate of agreement [39]. Given that this was meant to be a proof of principle, 90% of the recommended minimum (27 out of 30) was reached, and the results were consistent with part 1 of the study; it is sufficient to discuss trends but not confirm study findings. This study comprised a heterogenous sample where all participants had intermittent neck pain problems, but only some participants had pain in multiple regions within their spine. Future studies should administer the SMD-Q to participants with similar presentations of spinal dysfunction, to confirm study findings. As this study focused on assessing the reliability of the subsequent iterations of the SMD-Q, exploratory factor analysis, and ceiling and floor effects were not performed. Those analyses are more relevant for validating the structure and range of the questionnaire, which should be addressed in future studies as the reliability of the SMD-Q becomes more established.

## 5. Conclusions

The second and third iterations of the SMD-Q appear to reliably assess the impact of subclinical neck pain on constructs of SMI and multi-modal SMI, particularly when analyzing total scores. The phrasing of the initial portion of the questions with “in a typical/usual week” resulted in a greater consistency and reliability in total scores with a subclinical population who exhibit varying degrees of sensorimotor dysfunction. The use of either “over the past week” or “in a typical/usual week” is appropriate, but it may be worthwhile to test with a larger sample size and a population that has more congruent sensorimotor disturbances.

## Figures and Tables

**Table 1:** Descriptive and pain characteristics of the sample for Part 1 of the study.

	SCNP
Number of Participants	51
Biological Sex Ratio (F:M)	32:19
Age (years; mean ± SD)	21.17 ± 2.66
Duration/Onset of Pain (months)	25.77 ± 28.61
Pain VAS (cm)	
Baseline	28.16 ± 17.93
Follow-Up	24.35 ± 20.82
Von Korff Chronic Pain Grade Scale	
Score	45.19 ± 15.85
Disability Points	0.69 ± 0.99
	n
Pain Grade Classification	
Grade 0	0
Grade I	30
Grade II	16
Grade III	5
Grade IV	0
Clinical Characteristics	n
Region of Spinal Pain	
Neck	51
Upper Back	32
Mid Back	28
Low Back	34
Buttock	11

**Table 2:** Constructs of items and the corresponding weighted kappa statistics and confidence interval.

Construct of Items *	K_w_	95% CI
Item 10: Uni-sensory processing of visual stimuli	0.207	(−0.138, 0.551)
Item 8: Motor performance	0.230	(−0.160, 0.619)
Item 6: Failed during grasping motor tasks	0.234	(−0.092, 0.574)
Item 5: Missed during reaching motor tasks	0.303	(−0.019, 0.624)
Item 9: Uni-sensory processing of auditory stimuli	0.380	(0.153, 0.608)
Item 2: Hand–eye coordination	0.476	(0.324, 0.628)
Item 1: Postural control	0.510	(0.217, 0.802)
Item 11: Bombardment of sensory stimuli	0.581	(0.399, 0.763)
Item 7: Misstepped	0.605	(0.383, 0.826)
Item 12: MMI	0.634	(0.393, 0.875)
Item 3: Head and neck proprioception	0.682	(0.427, 0.938)
Item 4: Full-body proprioception (excluding head and neck)	0.700	(0.553, 0.847)
Total Score	0.820	(0.730, 0.911)

* Items are presented and ordered according to the K_w_ cut-offs.

**Table 3:** Descriptive and pain characteristics of the sample for part 2 of the study.

	SCNP
Number of Participants	27
Biological Sex Ratio (F:M)	20:7
Age (years; mean ± SD)	21.89 ± 2.81
Duration/Onset of Pain (months)	30.43 ± 36.16
Pain VAS (cm)	
Baseline	29.11 ± 13.18
Follow-Up	34.18 ± 22.20
Von Korff Chronic Pain Grade Scale	
Score	46.17 ± 19.63
Disability Points	1.33 ± 1.78
	n
Pain Grade Classification	
Grade 0	0
Grade I	15
Grade II	6
Grade III	4
Grade IV	2
Clinical Characteristics	n
Region of Spinal Pain	
Neck	27
Upper Back	20
Mid Back	15
Low Back	19
Buttock	4

**Table 4:** Weighted kappa statistics and confidence interval for the total score of the SMD-Q.

	K_w_		95% CI	
	“Over the past Week”	“In a typical/usual week”	“Over the past Week”	“In a typical/usual week”
Total	0.74	0.830	(0.589, 0.884)	(0.652, 1.008)

## Data Availability

The data can be made available by the corresponding author upon reasonable request. The data are not publicly available due to privacy and ethical restrictions.

## Appendix A. The Sensory–Motor Dysfunction Questionnaire (e.g., Questions About Your Brain-Body Communication)

This questionnaire seeks to determine to what degree your spinal dysfunction may be affecting how you process sensory information and therefore how it is affecting your function.

**Instructions:** Please answer **ALL** the scales, by choosing the option that is most applicable to you, in the **past week**.

**Table:**

	Never/Rarely When Performing This Task/Action (<1 Day)	Some or Little of the Time When Performing This Action/Task (1–2 Days)	Often or a Moderate Amount of Time When Performing This Action/Task (3–4 Days of the Week)	Most or All the Time When Performing This Action/Task (5 or More Days of the Week)
**Question 1:** Over the past week, on average, how often did you have problems with your physical balance (i.e., frequent loss of balance or unsteadiness or feel like you might fall while walking, running or standing still, etc.)?	☐	☐	☐	☐
**Question 2:** Over the past week, on average, how often did you have hand–eye coordination problems (e.g., several typos when typing on your phone or computer keyboard, several mistakes when playing an instrument while reading sheet music, reading and miswriting, misput key into door lock to unlock the door, making small mistakes when playing a sport with the hands, etc.)?	☐	☐	☐	☐
**Question 3:** Over the past week, how often have you accidentally bumped your head into things (i.e., hitting the top of your head when getting out of the car, bumping your head into a kitchen cupboard, etc.)?	☐	☐	☐	☐
**Question 4:** Over the past week, how often have you bumped into people or objects/things (i.e., table, wall, chair or leg of a chair, etc.) with other parts of your body?	☐	☐	☐	☐
**Question 5:** Over the past week, how often have you missed when you reached for an object without looking (e.g., phone, water bottle, cup, pen, book, kitchen tools, keys, etc.)?	☐	☐	☐	☐
**Question 6:** Over the past week, how often did you fail to pick something up that you initially dropped (i.e., slipped out of your hands or butter fingers, etc.)?	☐	☐	☐	☐
**Question 7:** Over the past week, how often have you missed when you had to use your leg or foot (i.e., misplaced or awkward step when walking or walking up/down the staircase or stepping up to a chair or stool, missed when kicking a ball or putting your foot in slip-on shoes, pushing against the gas pedal instead of the brake pedal, etc.)?	☐	☐	☐	☐
**Question 8:** Over the past week, how often have struggled to perform a movement you normally do well (e.g., missed when hitting or catching a ball, tripped while walking or running, struggled with a musical instrument you normally play well, pushing against the gas or brake pedal too hard or soft, biting the side of your mouth, lip or tongue while eating, etc.)?	☐	☐	☐	☐
**Question 9:** Over the past week, how often have you had trouble recognizing familiar sounds that you normally recognize very quickly (e.g., mishearing words or phrases when someone is speaking to you or mishearing lyrics of a familiar song)?	☐	☐	☐	☐
**Question 10:** Over the past week, how often have you had trouble recognizing familiar objects, places or people that you normally recognize very quickly (e.g., recognizing that a cat ran past as opposed to just a fast-moving object or recognizing familiar face(s) when you walk past them at the grocery store, in the hallways at work or school, or recognize the place that you frequently pass by, etc.)?	☐	☐	☐	☐
**Question 11:** Over the past week, how often have you been having difficulties concentrating in situations where there is a lot of background noise (e.g., people coughing, side conversations around you, driving while listening to music and passengers having a conversation with you, etc.)?	☐	☐	☐	☐
**Question 12:** Over the past week, how often have you had difficulties performing tasks that require you to combine information from more than one sense (sound, sight, smell, taste, etc.) at the same time (i.e., you aren’t as fast at combining sight and sound information from traffic so you have difficulty gauging how much time you have to cross the street, or during online gaming; or food is not smelling and tasting as it used to, or you are not as accurate at judging the space available to pass through an opening so you bump into things, etc.)?	☐	☐	☐	☐

## Appendix B. The Sensory–Motor Dysfunction Questionnaire (e.g., Questions About Your Brain-Body Communication)

This questionnaire seeks to determine to what degree your spinal dysfunction may be affecting how you process sensory information and therefore how it is affecting your function.

**Instructions:** Please answer **ALL** the scales, by choosing the option that is most applicable to you, in a **typical/usual week**.

**Table:**

	Never/Rarely When Performing This Task/Action (<1 Day)	Some or Little of the Time When Performing This Action/Task (1–2 Days)	Often or a Moderate Amount of Time When Performing This Action/Task (3–4 Days of the Week)	Most or All the Time When Performing This Action/Task (5 or More Days of the Week)
**Question 1:** In a typical/usual week, how often did you have problems with your physical balance (i.e., frequent loss of balance or unsteadiness or feel like you might fall while walking, running or standing still, etc.)?	☐	☐	☐	☐
**Question 2:** In a typical/usual week, how often did you have hand–eye coordination problems (e.g., several typos when typing on your phone or computer keyboard, several mistakes when playing an instrument while reading sheet music, reading and miswriting, misput key into door lock to unlock the door, making small mistakes when playing a sport with the hands, etc.)?	☐	☐	☐	☐
**Question 3:** In a typical/usual week, how often have you accidentally bumped your head into things (i.e., hitting the top of your head when getting out of the car, bumping your head into a kitchen cupboard, etc.)?	☐	☐	☐	☐
**Question 4:** In a typical/usual week, how often have you bumped into people or objects/things (i.e., table, wall, chair or leg of a chair, etc.) with other parts of your body?	☐	☐	☐	☐
**Question 5:** In a typical/usual week, how often have you missed when you reached for an object without looking (e.g., phone, water bottle, cup, pen, book, kitchen tools, keys, etc.)?	☐	☐	☐	☐
**Question 6:** In a typical/usual week, how often did you fail to pick something up that you initially dropped (i.e., slipped out of your hands or butter fingers, etc.)?	☐	☐	☐	☐
**Question 7:** In a typical/usual week, how often have you missed when you had to use your leg or foot (i.e., misplaced or awkward step when walking or walking up/down the staircase or stepping up to a chair or stool, missed when kicking a ball or putting your foot in slip-on shoes, pushing against the gas pedal instead of the brake pedal, etc.)?	☐	☐	☐	☐
**Question 8:** In a typical/usual week, how often have struggled to perform a movement you normally do well (e.g., missed when hitting or catching a ball, tripped while walking or running, struggled with a musical instrument you normally play well, pushing against the gas or brake pedal too hard or soft, biting the side of your mouth, lip or tongue while eating, etc.)?	☐	☐	☐	☐
**Question 9:** In a typical/usual week, how often have you had trouble recognizing familiar sounds that you normally recognize very quickly (e.g., mishearing words or phrases when someone is speaking to you or mishearing lyrics of a familiar song)?	☐	☐	☐	☐
**Question 10:** In a typical/usual week, how often have you had trouble recognizing familiar objects, places or people that you normally recognize very quickly (e.g., recognizing that a cat ran past as opposed to just a fast-moving object or recognizing familiar face(s) when you walk past them at the grocery store, in the hallways at work or school, or recognize the place that you frequently pass by, etc.)?	☐	☐	☐	☐
**Question 11:** In a typical/usual week, how often have you been having difficulties concentrating in situations where there is a lot of background noise (e.g., people coughing, side conversations around you, driving while listening to music and passengers having a conversation with you, etc.)?	☐	☐	☐	☐
**Question 12:** In a typical/usual week, how often have you had difficulties performing tasks that require you to combine information from more than one sense (sound, sight, smell, taste, etc.) at the same time (i.e., you aren’t as fast at combining sight and sound information from traffic so you have difficulty gauging how much time you have to cross the street, or during online gaming; or food is not smelling and tasting as it used to, or you are not as accurate at judging the space available to pass through an opening so you bump into things, etc.)?	☐	☐	☐	☐

**Instructions:** Please answer **ALL** the scales, by choosing the option that is most applicable to you, in the **past week**.

**Table:**

	Never/Rarely When Performing This Task/Action (<1 Day)	Some or Little of the Time When Performing This Action/Task (1–2 Days)	Often or a Moderate Amount of Time When Performing This Action/Task (3–4 Days of the Week)	Most or All the Time When Performing this Action/Task (5 or More Days of the Week)
**Question 1:** Over the past week, on average, how often did you have problems with your physical balance (i.e., frequent loss of balance or unsteadiness or feel like you might fall while walking, running or standing still, etc.)?	☐	☐	☐	☐
**Question 2:** Over the past week, on average, how often did you have hand–eye coordination problems (e.g., several typos when typing on your phone or computer keyboard, several mistakes when playing an instrument while reading sheet music, reading and miswriting, misput key into door lock to unlock the door, making small mistakes when playing a sport with the hands, etc.)?	☐	☐	☐	☐
**Question 3:** Over the past week, how often have you accidentally bumped your head into things (i.e., hitting the top of your head when getting out of the car, bumping your head into a kitchen cupboard, etc.)?	☐	☐	☐	☐
**Question 4:** Over the past week, how often have you bumped into people or objects/things (i.e., table, wall, chair or leg of a chair, etc.) with other parts of your body?	☐	☐	☐	☐
**Question 5:** Over the past week, how often have you missed when you reached for an object without looking (e.g., phone, water bottle, cup, pen, book, kitchen tools, keys, etc.)?	☐	☐	☐	☐
**Question 6:** Over the past week, how often did you fail to pick something up that you initially dropped (i.e., slipped out of your hands or butter fingers, etc.)?	☐	☐	☐	☐
**Question 7:** Over the past week, how often have you missed when you had to use your leg or foot (i.e., misplaced or awkward step when walking or walking up/down the staircase or stepping up to a chair or stool, missed when kicking a ball or putting your foot in slip-on shoes, pushing against the gas pedal instead of the brake pedal, etc.)?	☐	☐	☐	☐
**Question 8:** Over the past week, how often have struggled to perform a movement you normally do well (e.g., missed when hitting or catching a ball, tripped while walking or running, struggled with a musical instrument you normally play well, pushing against the gas or brake pedal too hard or soft, biting the side of your mouth, lip or tongue while eating, etc.)?	☐	☐	☐	☐
**Question 9:** Over the past week, how often have you had trouble recognizing familiar sounds that you normally recognize very quickly (e.g., mishearing words or phrases when someone is speaking to you or mishearing lyrics of a familiar song)?	☐	☐	☐	☐
**Question 10:** Over the past week, how often have you had trouble recognizing familiar objects, places or people that you normally recognize very quickly (e.g., recognizing that a cat ran past as opposed to just a fast-moving object or recognizing familiar face(s) when you walk past them at the grocery store, in the hallways at work or school, or recognize the place that you frequently pass by, etc.)?	☐	☐	☐	☐
**Question 11:** Over the past week, how often have you been having difficulties concentrating in situations where there is a lot of background noise (e.g., people coughing, side conversations around you, driving while listening to music and passengers having a conversation with you, etc.)?	☐	☐	☐	☐
**Question 12:** Over the past week, how often have you had difficulties performing tasks that require you to combine information from more than one sense (sound, sight, smell, taste, etc.) at the same time (i.e., you aren’t as fast at combining sight and sound information from traffic so you have difficulty gauging how much time you have to cross the street, or during online gaming; or food is not smelling and tasting as it used to, or you are not as accurate at judging the space available to pass through an opening so you bump into things, etc.)?	☐	☐	☐	☐
